# Impact of Wall Material Composition (Maltodextrin vs. Inulin vs. Nutriose) and Emulsion Preparation System (Nano- vs. Microemulsion) on Properties of Spray-Dried Linseed Oil

**DOI:** 10.3390/molecules30010171

**Published:** 2025-01-04

**Authors:** Dorota Ogrodowska, Iwona Zofia Konopka, Grzegorz Dąbrowski, Beata Piłat, Józef Warechowski, Fabian Dajnowiec, Małgorzata Tańska

**Affiliations:** 1 Department of Food Plant Chemistry and Processing, Faculty of Food Sciences, University of Warmia and Mazury, Plac Cieszyński 1, 10-726 Olsztyn, Poland; dorota.ogrodowska@uwm.edu.pl (D.O.); iwona.konopka@uwm.edu.pl (I.Z.K.); grzegorz.dabrowski@uwm.edu.pl (G.D.); beata.pilat@uwm.edu.pl (B.P.); 2 Department of Process Engineering, Equipment and Food Biotechnology, Faculty of Food Sciences, University of Warmia and Mazury, Oczapowskiego 7, 10-719 Olsztyn, Poland; jozef.w@uwm.edu.pl (J.W.); fabian.dajnowiec@uwm.edu.pl (F.D.)

**Keywords:** linseed oil, encapsulation, inulin, maltodextrin, nutriose, nanoemulsion, oxidative stability, powder properties

## Abstract

The aim of this study was to compare the functional properties of linseed oil powders made of three types of wall material (OSA starch + maltodextrin, OSA starch + nutriose, and OSA starch + inulin) and two types of emulsion phases (micro- and nanoemulsion). For these independent variables, the properties of the prepared emulsions (flow curves and viscosity) and the resulting powders (encapsulation efficiency, particle size distribution, water activity, bulk and tapped density, Carr’s index, color parameters, and thermal stability) were determined. The results showed that emulsion viscosity and most powder properties were affected by the emulsion type. All emulsions demonstrated Newtonian-like behavior, with viscosity values ranging from 29.07 to 48.26 mPa·s. The addition of nutriose induced the most significant variation in this parameter, with nanoemulsification leading to a 1.6-fold increase in viscosity compared to microemulsification. The application of nanoemulsification to prepare the emulsions prior to spray-drying resulted in powders with lower surface oil content (by 78.8–88.5%), tapped density (by 1.7–14.2%), and Carr’s index (by 7.6–14.0%), as well as higher encapsulation efficiency (by 5.9–17.0%). The decreased oxidative stability (by 30.9–51.1%) of powders obtained from nanoemulsified emulsions was related to 4.7–15.9-fold lower surface oil content. Powders produced using inulin as the wall material had the smallest and most uniform particle sizes, showing minimal variation between powders derived from nano- and microemulsified emulsions.

## 1. Introduction

Alpha-linolenic acid (ALA) is rarely found as the primary component of plant oils. Currently, two plants of economic importance serve as key sources of ALA-rich oils: linseed (*Linum usitatissimum* L.) and chia seed (*Salvia hispanica* L.). The seeds of these plants can contain up to 60% ALA among their total fatty acids [1,2,3,4]. Linseed is considered the superior source of ALA-rich oil due to its higher global production compared to chia seeds. For instance, global linseed production reached 3,973,932 tons in 2022 [5], while chia seed production was 60,000 tons in 2017, with projections up to 100,000 tons by 2027 [6].

After ingestion, ALA is incorporated into body lipid pools and subsequently metabolized endogenously through desaturation, elongation, and peroxisomal oxidation to produce eicosapentaenoic acid (EPA) and docosahexaenoic acid (DHA) [7,8,9]. Although the conversion of ALA to EPA and DHA is very limited, particularly for DHA [9,10], plant oils rich in ALA are potential precursors of long-chain polyunsaturated fatty acids (PUFAs) in vegan and vegetarian diets [11,12]. PUFAs, especially omega-3 representants, are well-documented to have positive effects on human health. The Food and Agriculture Organization (FAO) and World Health Organization (WHO) recommend a daily intake of 0.25–2 g EPA + DHA for the prevention of various diseases [13]. Despite the low conversion rate of ALA to EPA + DHA (0.3–10%) in the human body (more data in the work from Watabe et al. [10]), the high ALA content in linseed oil can maintain the optimal level of Omega-3 Index [14].

Unfortunately, ALA can be easily oxidized through photooxidation, auto-oxidation, and enzyme-assisted reactions [15]. Co-supplementation with antioxidants such as vitamin E can mitigate PUFA oxidation and stabilize native structures of unsaturated fatty acids [16]. Encapsulation is another effective method for protecting PUFAs by shielding them from oxygen and also hiding any unwanted flavors from raw materials. Currently, this technique is increasingly utilized for incorporating unstable substances, such as vitamins, lipids, pigments, flavors, proteins, or probiotics, into food matrices [17,18].

Micro- and nano-encapsulation are widely used in the food industry to encapsulate oils. Microencapsulation involves loading oil droplets into microparticles (>1 μm) [19]. Microemulsions are transparent, thermodynamically stable systems prepared with relatively low-energy inputs [20]. Reducing microparticle size to below 1 μm forms nanoencapsulation systems, enhancing the surface-to-volume ratio. Nanoemulsions are submicron (20–500 nm) isotropic colloidal dispersions that appear optically clear or translucent. Nanoemulsification offers some superior advantages, such as higher bioavailability, solubility, and absorption [21]. More data on the comparison between nano- and microemulsion preparation and properties can be found in the review by Garavand et al. [20]

Nanoprocessing helps to improve the properties of food products and makes bioactive food ingredients more bioavailable and soluble in water [22]. Nanoemulsions, due to their small size, quantum size effect, and high surface-to-volume ratio, have also been found to improve lipid absorption in the gastrointestinal tract. For example, Hu et al. [23] demonstrated increased fatty acid levels in plasma and liver tissues of rats administered nanoemulsified perilla oil. Additionally, nanoemulsions can enhance crude oil recovery by 70%, while microemulsion flooding reduces residual oil saturation by approximately 30% compared to water flooding, achieving total oil recovery of 80% [24]. Spray drying is still a crucial technique to convert oil emulsion to powders. It involves atomizing a liquid or emulsion into hot air or inert gas, resulting in particles with a moderately narrow size distribution depending on the feed material and operating conditions (more data in the work of Deshmukh et al. [25]).

Maltodextrin and octenyl succinic anhydride (OSA) starch (both with a caloric value of approximately 4 kcal/g [26]) are widely used wall materials in encapsulation processes [27,28,29]. However, there is ongoing research to identify reduced-calorie alternatives. Our previous study showed that two commercial soluble fibers, nutriose and inulin, can replace maltodextrin [30] during the encapsulation process. Nutriose contains α-D 1,4-, 1,6-, 1,2-, and 1,3-glycosidic bonds at ratios of 41%, 32%, 13%, and 14%, respectively, and provides 1.7 kcal/g [31]. In contrast, inulin, a fructan consisting of fructosyl units linked by β-D (2,1) glycosidic bonds, has well-established prebiotic properties and a caloric value of 1.5 kcal/g [32]. Due to the fact that the ratio of wall material to lipid core is often 2 to 1 or higher [33], reducing the caloric value of the powder is desirable for health reasons.

The aim of the present study is to compare the functional properties of linseed oil powders prepared using three wall material combinations (OSA starch + maltodextrin, OSA starch + nutriose, and OSA starch + inulin) and two types of emulsion phase preparations (micro- and nanoemulsion). For these independent variables, the viscosity of the prepared emulsions and characteristics of the obtained powders (encapsulation efficiency, particle size, density, color, and thermal stability) were determined. The novelty of this publication is the comparison of powders obtained using two types of emulsions, which is rarely presented in other studies. The use of inulin and nutriose for the production of lipid powders is also explored relatively little in comparison to maltodextrin, OSA starch, and various proteins.

## 2. Results and Discussion

### 2.1. Effect of Components and Preparation System on Emulsion Characteristics

**Figure 1:**
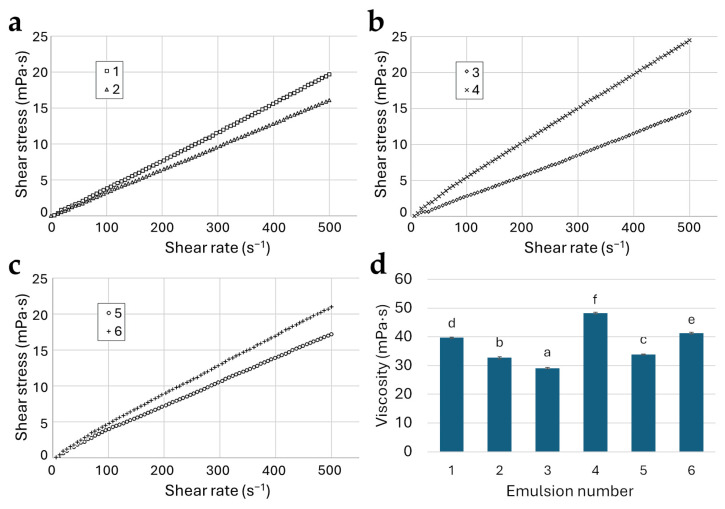
Flow curves (**a**–**c**) and viscosity (**d**) of linseed oil emulsions prepared with maltodextrin (1,2), nutriose (3,4), and inulin (5,6) using micro- (1,3,5) and nanoemulsification (2,4,6), plotted with shear stress over shear rate. ^a–f^ bars (mean ± standard deviation) in subfigure d marked with different letters indicate significant differences (*p* < 0.05).

Figure 1 presents the flow characteristics and viscosity of the prepared emulsions. The results show a linear relationship between shear stress and shear rate (Figure 1a–c) and confirm that all emulsions exhibited Newtonian-like behavior, with viscosity values ranging from 29.07 to 48.26 mPa·s (Figure 1d). A two-way ANOVA revealed that viscosity was significantly influenced (*p* < 0.05) by both the preparation method and the emulsion composition (Figure S1). Theoretically, the viscosity of an emulsion depends mainly on the volume fraction and viscosity of dispersed and continuous phases and temperature, along with several minor effects, such as shear rate, average droplet size, and droplet size distribution [34]. Among the constant variables used in the current study, droplet size values should be decisive. It has been previously confirmed that fine emulsion droplets produced, for example, by nanoemulsification tend to have higher viscosity than the corresponding coarse emulsions [35]. Newtonian emulsions exhibit constant viscosity under varying shear rates, e.g., during the atomization process. Breaking emulsion droplets during atomization enhances surface oil content [36]. It was found that emulsions containing nutriose exhibited the widest range of viscosity values, encompassing both the lowest and highest measurements. In contrast, emulsions prepared with maltodextrin and inulin demonstrated less variation. In our opinion, the observed variability may be related, among other things, to the degree of polymerization (DP) of the polysaccharides used. For the maltodextrin, DP was 7–8 (value calculated from dextrose equivalent), while typical values for inulin and nutriose are 2–60 [37] and 12–25 [38], respectively. We believe that further studies should focus on comparing inulin, nutriose, and maltodextrin preparations with similar degrees of polymerization.

The viscosity results for emulsions prepared by microemulsification correspond with the range (approximately 20–75 mPa·s) reported by Carneiro et al. [39] for microemulsions containing flaxseed (linseed) oil, maltodextrin, and OSA. In contrast, Sharif et al. [40] determined significantly higher viscosity levels (>74 mPa·s) for nanoemulsions containing linseed oil, eugenol, and OSA starch.

### 2.2. Effect of Emulsion Components and Preparation System on Oil Encapsulation Efficiency

**Figure 2:**
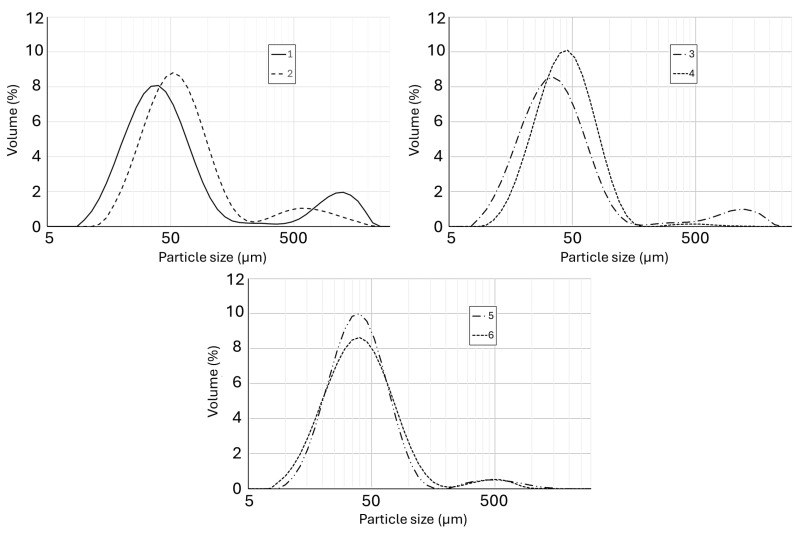
Particle size distribution (by volume) of powders from linseed oil emulsions prepared with maltodextrin (1,2), nutriose (3,4), and inulin (5,6) using micro- (1,3,5) and nanoemulsification (2,4,6).

The surface oil content and encapsulation efficiency are presented in Table 1. The surface oil content ranged from 0.29% to 4.62%, while encapsulation efficiency ranged from 84.62% to 99.03%. A two-way analysis of variance showed a significant (*p* < 0.05) impact of the emulsification system on these parameters (Figure S1). Generally, powders prepared using nanoemulsions were characterized by the same level of values (no significant differences were found according to emulsion composition), with higher encapsulation efficiency and lower surface oil content compared to powders made from microemulsions. The low oil content on the powder surface was due to the presence of relatively small drops in the emulsion, which did not disintegrate during the atomization process [36,41]. For powders prepared using microemulsions, the highest encapsulation efficiency was observed for the composition with inulin (93.08%), while the lowest was observed for nutriose in formulation (84.62%). At the same time, the surface oil contents were 2.08% and 4.62%, respectively. El-Messery et al. [42] obtained a significantly lower (51.9–58.2%) encapsulation efficiency for krill oil in the nanoemulsion system. In general, increasing emulsions’ viscosity up to an optimal level enhances encapsulation efficiency and oil retention [43,44]. As shown in Figure 2, the viscosity of emulsions made with nutriose varied significantly between preparation systems, with nanoemulsion exhibiting higher viscosity. According to Stoke’s law, higher viscosity in an emulsion contributes to greater stability [45]. For the prepared microemulsions, encapsulation efficiency was influenced by the type of carbohydrate used. According to manufacturer specifications, nutriose is a low-viscosity fiber [31]. Similarly, inulin, at a 10% concentration and temperatures of 25–40 °C, produces fluids with viscosities of 2.72–2.66 mPa·s [46]. These values indicate that both fibers are practically non-viscous, as the viscosity of water at 21 °C is approximately 1 mPa·s [47]. Maltodextrin, at the concentration used in this study, also produces fluids with viscosities comparable to those of inulin and nutriose at room temperature [48]. This suggests that the primary component contributing to the viscous continuous phase was OSA starch. In our previous research, an emulsion prepared with this carbohydrate as the main component (3:1 mass ratio) exhibited approximately 5–6 times higher viscosity than emulsions made with a predominance of inulin or nutriose [30]. Additionally, it is worth mentioning that the polysaccharides used for encapsulation in the current study have different thermal properties, for example, glass transition temperature (this temperature represents the narrow temperature range of the transition of polymers from a hard and brittle glass into a softer, rubbery state [49]. The glass transition temperature (measured at the same water content/water activity and temperature) depends on the polysaccharide polymerization index [50], and among the wall materials used, it was the highest for nutriose (180 °C for dry material), while for maltodextrin and inulin, it was close to 150 °C and 120 °C, respectively (data also apply to dry material) [31,51,52]. In the case of hydrated compounds, the glass transition temperature decreased, and in this state, the viscosity of the emulsion increased rapidly [53]. This points out that, for the spray-drying conditions used (inlet air temperature in the range of 123–129 °C), maltodextrin and inulin probably had better conditions for the glass transition process, which affected the final emulsion’s viscosity and encapsulation efficiency.

### 2.3. Effect of Emulsion Components and Preparation System on Powder Dimension Characteristics

The particle sizes of linseed oil powders were characterized by the parameters d(0.1), d(0.5), d(0.9), D[3, 2], and D[4, 3] (Table 2). Powders formulated with maltodextrin generally exhibited larger particle sizes and broader size distributions, as evidenced by higher D[4, 3] and d(0.9) values. Specifically, the D[4, 3] for powder derived from nanoemulsified oil with maltodextrin was 100.9 μm, which exceeded the values observed for powders with nutriose (54.7 μm) and inulin (57.1 μm). The application of nutriose resulted in powders with intermediate particle sizes. For example, the d(0.9) of powder from a nanoemulsified emulsion containing nutriose was 81.7 μm, significantly smaller than the 144.8 μm observed for its maltodextrin counterpart. However, powders containing this carbohydrate were characterized by particle sizes significantly influenced by the emulsion preparation system. In contrast, the use of inulin produced powders with the smallest and most uniform particle sizes, exhibiting minimal variation between powders derived from nanoemulsified and microemulsified emulsions. The d(0.5) values for inulin-based powders were closely aligned, measuring 36.4 μm and 37.1 μm for powders obtained from micro- and nanoemulsions, respectively. A two-way analysis of variance showed significant (*p* < 0.05) effects of both independent variables (emulsion composition and emulsification system) and their interaction on all parameters characterizing particle size of powders (Figure S1).

Significantly lower D[4, 3] values (12–59 μm) have been reported for powders from spray-dried linseed oil microemulsions containing maltodextrin with Arabic gum [54], maltodextrin with plant proteins (pea and rice mixture) [55], maltodextrin with pectin and wax [56], OSA starch combined with trehalose [57], and OSA starch with maltodextrin [39]. De Barros Fernandes et al. [58] showed that formulations using inulin and maltodextrin resulted in D[4, 3] values averaging 12.1 μm. The authors also reported low Span values (1.75–2.45) consistent with those observed in this study, indicating a homogeneous particle size distribution.

The obtained powders presented a bimodal distribution of particle sizes (Figure 2). The first fraction constituted the main part of the powders and consisted of particles with sizes of 6–200 μm. The second fraction was a marginal one exhibiting large particles (>300 μm). The emulsion preparation system had only a minor impact on particle size when inulin was used. However, this factor significantly affected the particle size distribution in the case of maltodextrin’s and nutriose’s presence in the emulsion formulations. Nanoemulsification of the emulsions resulted in a reduced proportion of larger particles in these powders. Additionally, the maximum peaks showed a slight shift to the right, indicating an increase in the median particle size, as reflected by the d(0.5) value (Table 2). Notably, for nutriose, the powder particles showed a distribution close to monomodal. The proportion of the powder fractions directly affected the value of the specific surface area (SSA). This phenomenon is particularly visible in the case of powders 1–4. Powders 1 and 3, with higher proportions of smaller particles (the first peak shifted to the left), were characterized by increased SSA values (Table 2). In contrast, powders 5 and 6, with similar particle size distributions (Figure 2), had comparable SSA values (Table 2). In the study conducted by Sotelo-Bautista et al. [59], powders obtained by spray drying (with the use of Büchi apparatus) presented a trimodal distribution. The authors also used as one of the variants a coating consisting of maltodextrin and OSA starch. On the other hand, Elik et al. [56] reported a monomodal distribution for powders also prepared on the mini spray dryer. Taking into account our results and the data presented in the literature, it should be concluded that the type of equipment used for drying has a strong impact on the particle distribution.

### 2.4. Effect of Emulsion Components and Preparation System on Powders Water Activity, Density, Carr’s Index, and Color

Foods, mostly in the form of powders, are very stable at water activity (a_w_) levels close to the monomolecular moisture content, where the rate for lipid peroxidation is lowest. Besides a_w_, the phenomenon of glass transition could be applied as an integrated approach to determine food stability. Maximum food stability occurs when the storage temperature is below the glass transition temperature, i.e., food is in the glassy state [60]. In our study, the a_w_ value was similar in all powders (range 0.26–0.31) (Table 3). Tatar et al. [61] stated that a_w_ values less than 0.30 are normally considered to ensure product stability, and in their study, a_w_ values were in the range of 0.05–0.08 for microcapsules with hemicellulose and gum Arabic as a coating material. In turn, Lacerda et al. [62] reported a_w_ to fall into the range of 0.252–0.484, where the lowest value was for the sample containing a high proportion of inulin.

Parameters characterizing the flow properties of powders (bulk and tapped densities, and Carr’s index) are presented in Table 3. These parameters provide critical insights into the behavior of powders during handling, processing, and storage [63]. Bulk (aerated) density refers to the powder’s mass per unit volume as poured, while tapped (packaged) density accounts for the densification that occurs after tapping. Both densities influence packing efficiency and flowability [64]. All powders obtained were characterized by low bulk density, ranging from 0.248 to 0.289 g/cm³. This parameter was significantly influenced by the emulsion composition (*p* < 0.05). Powders containing inulin demonstrated higher bulk density, with values of 0.267 and 0.289 g/cm³ for microemulsification and nanoemulsification, respectively. Literature reports generally indicate higher bulk density values (0.34–0.39 g/cm^3^) for linseed oil powders prepared by spray-drying of microemulsified emulsions [57,65]. In a study conducted by Domian et al. [57], applied carbohydrate components (trehalose, wheat dextrin soluble fiber) had no significant effect on the obtained results, but the powders with pea protein addition were characterized by a significantly higher bulk density compared to powders with soy protein.

Tapped density values varied over a considerably wider range, from 0.417 to 0.533 g/cm³. It was observed that sample 4 (powder prepared from nanoemulsion with nutriose) had the lowest tapped density, with a statistically significant difference compared to the other samples. Additionally, it was found that the emulsification system significantly (*p* < 0.05) differentiated powders containing nutriose. Microemulsification resulted in a powder with a tapped density more than 60% higher than that of the powder obtained from the nanoemulsion.

The Carr’s index was used to evaluate the flow behavior of the powders. A higher Carr’s index reflected enhanced powder compressibility and decreased flowability [65]. For the prepared powders, Carr’s index values ranged from 40.46 to 49.96, respectively (Table 3). This parameter varied significantly based on the emulsification system (*p* < 0.05), with additional differences found among the carbohydrates used in the emulsion preparation (Figure S1). Overall, nanoemulsification consistently improved flow properties, with the most significant improvements noted in samples prepared with nutriose. In contrast, microemulsification generally resulted in powders with higher cohesiveness and poorer flowability, particularly with maltodextrin and inulin. A Carr’s index value (40.56) was presented by Perrechil et al. [55] for powders of flaxseed oil with OSA starch as a coating material. In turn, in powders where plant proteins were used, Carr’s index was below the value of 40 [65].

Table 4 presents the results of color measurements for the produced powders. It was observed that the powders, regardless of the emulsion composition and preparation, exhibited similar lightness values (L* ranging from 96.66 to 98.65). Powders containing nutriose, however, showed slightly higher lightness. In this case, the L* parameter was significantly (*p* < 0.05) influenced only by the interaction between the studied factors (Figure S1). The powder samples also demonstrated limited variation in the a* and b* parameters. Sample 5 (powder prepared from microemulsion with inulin) differed significantly from the others, with a higher contribution of green (a* = −2.21) and yellow (b* = 8.04) tones. For the remaining samples, the a* and b* values ranged from −1.87 to −1.61 and from 5.07 to 6.58, respectively. Furthermore, it was observed that, for the a* parameter, the interaction between the studied factors had a stronger influence (*p* < 0.05), while the emulsion preparation system had a less significant effect (*p* < 0.05). In contrast, for the b* parameter, both factors demonstrated a statistically significant (*p* < 0.05) influence, with the emulsification system appearing to have a greater impact. Differences in color for powders obtained with maltodextrin and inulin were also reported by Lacerda et al. [62]. The cited authors obtained powders with inulin characterized by a lower a* parameter and a higher b* parameter compared to powders with maltodextrin.

**Figure 3:**
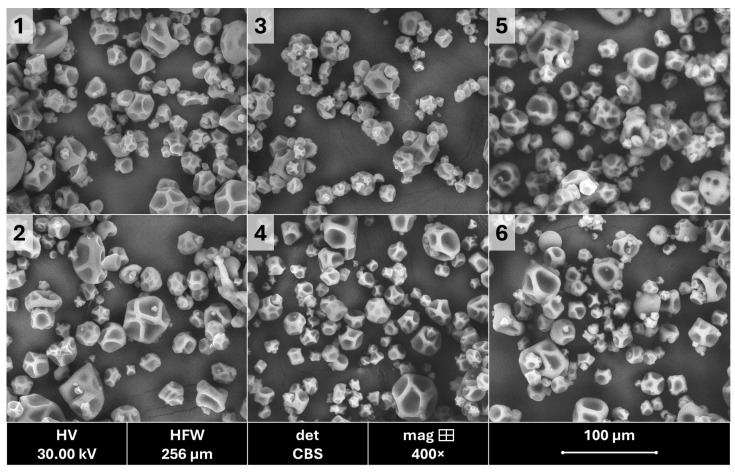
Images of powders from linseed oil emulsions prepared with maltodextrin (1,2), nutriose (3,4), and inulin (5,6) using micro- (1,3,5) and nanoemulsification (2,4,6).

The microstructures of the linseed oil-containing powders were analyzed using SEM, revealing particles with irregular shapes and sizes (Figure 3). Similar findings were reported by Jan et al. [66] for powders obtained through nanoemulsification. Domian et al. [67], who investigated the functional properties of linseed oil microencapsulated via spray drying, described powders containing soluble fiber (Nutriose FB06) as having irregular shapes with multiple indentations and craters, consistent with our results, as shown in Figure 3. Similarly, Elik et al. [56] observed particles with dents, though without cracks or pores. They attributed the wrinkled surface morphology to the high inlet drying air temperature, which causes rapid surface water evaporation.

Additionally, aggregation of microcapsules was observed in samples derived from emulsions prepared via microemulsification (samples 1, 3, and 5). This phenomenon may be attributed to the higher oil content on the powder surface (Table 1). This observation corresponds to the presence of larger particle fractions in these powders, as evident from the particle size distributions shown in Figure 2. A similar trend was reported by Sotelo-Bautista et al. [59].

### 2.5. Effect of Emulsion Components and Preparation System on the Powder Oxidative Stability

**Figure 4:**
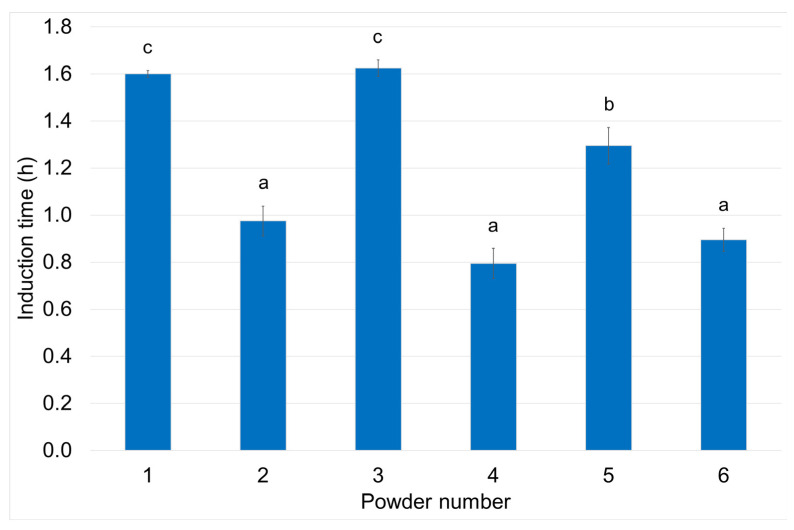
Oxidative stability (expressed as induction time at 110 °C) of powders from linseed oil emulsions prepared with maltodextrin (1,2), nutriose (3,4), and inulin (5,6) using micro- (1,3,5) and nanoemulsification (2,4,6). Different letters indicate significant differences (*p* < 0.05).

The oxidative stability of the linseed oil powders was analyzed using the Rancimat apparatus with the accelerated (110 °C) oxidation assay (Figure 4). This method measures the increase in conductivity of deionized water related to the increase in polar volatile compounds formed during oil oxidation in the water [68].

A two-way analysis of variance showed that both independent variables significantly (*p* < 0.05) influenced this powder property, although the effect of emulsion composition was notably less significant (Figure S1). The lowest values of IT were determined for all three samples produced using nanoemulsions, ranging from 0.78 to 0.98 h, with no significant differences attributable to emulsion composition. In contrast, powders produced from microemulsions exhibited significantly higher (*p* < 0.05) IT values (1.3–1.6 h), with the results further differentiated by the type of carbohydrate used (maltodextrin = nutriose > inulin).

While the IT results suggest lower oxidative stability for powders prepared using nanoemulsions, this should be considered alongside the approximately 5–16-fold lower surface oil content of these samples (Table 2). Carbohydrates used as wall components in the current study typically provide good oxygen barrier properties in spray-dried oil powders [69], and in this case, oxidation likely occurs predominantly at the surface. Given the small proportion of surface oil content (0.29–0.48% of the total encapsulated oil), the IT values likely reflect the oxidative stability of the residual surface lipids. Our results confirm the previously determined findings for encapsulated chia oil [70] and cod liver oil [71].

## 3. Materials and Methods

### 3.1. The Materials

Cold-pressed linseed oil was extracted from commercially available seeds from the local market in Olsztyn (Poland), using an IBG Monforts & Reiners, Komet CA59G (Mönchengladbach, Germany) laboratory screw press equipped with a 4 mm diameter nozzle. The pressing process was conducted at a temperature below 45 °C. The obtained oil samples were purified via centrifugation at 12,333× *g* using an Eppendorf 5810R centrifuge (Eppendorf AG, Hamburg, Germany) and subsequently utilized for emulsion preparing. The obtained oil was characterized by a high α-linolenic percentage, accounting for 54% of the total fatty acids, and demonstrated good quality, as confirmed by its low acid value (0.5 mg KOH/g) and peroxide value (0.95 mEq O_2_/kg). Maltodextrin (DE 18), Nutriose^®^ (soluble maize fiber), and octenyl succinic anhydride starch (OSA starch, waxy maize basis) were purchased from Roquette (Lestrem, France). Inulin, derived from chicory root, was purchased from Simpatiko sp. z o.o. (Łuze, Poland).

### 3.2. Emulsions Preparation and Spray-Drying

Two liters of emulsions containing 70% distilled water, 10% linseed oil, 10% OSA starch, and 10% of various carbohydrates, such as maltodextrin or soluble dietary fibers, were prepared. The components were mixed at 30 °C for 20 min using a Thermomix (Vorwerk Elektrowerke GmbH & Co. KG, Wuppertal, Germany) and then homogenized. Homogenization was conducted either in two stages, at 24 MPa (first step) and 4 MPa (second step), using a high-pressure laboratory valve homogenizer (Panda 2K, GEA Niro Soavi, Parma, Italy), or in a single stage at 150 MPa using a M110P F3 Microfluidizer™ with Interaction Chamber type F12Y (75 µm) and Auxiliary Processing Module type H30Z (200 µm) (Microfluidics International Corporation, Westwood, MA, USA). A total of six emulsions were prepared, and their characteristics are detailed in Table 5.

The emulsions were spray-dried using a Production Minor Spray Dryer (A/S Niro Atomizer, Copenhagen, Denmark) equipped with a disc spray system. The process parameters were selected based on our previous studies [30]. The inlet air temperature ranged from 123 °C to 129 °C, while the outlet temperature ranged from 72 °C to 78 °C. The feed flow rate was maintained at 77 mL/min and carefully monitored throughout the process.

### 3.3. Emulsions Viscosity Characteristic

The rheological properties of the emulsions were evaluated using a Rheolab QC rheometer (Anton Paar, Austria). A dynamic test was conducted in which the shear rate increased from 0 to 500 1/s. In this shear rate range, 80 points were measured at 5 s intervals. Measurements were conducted at a controlled temperature of 21 ± 0.5 °C.

### 3.4. Powders Characteristic

#### 3.4.1. Encapsulation Efficiency Analysis

The content of non-encapsulated oil (surface oil) in the powders was determined by washing the samples three times with hexane, following the method described by Liu et al. [72] with modifications. The analysis was carried out using hexane (50 mL), which was added to 5 g of powder in a 250 mL flask, and the sample was shaken for 5 min. The slurry was then filtered through a filter paper, and each filter paper with solid particles was washed of hexane. The filtrate caught into a round-bottom flask was then mounted in the rotary evaporator (Büchi Labortechnik AG, Flawil, Switzerland) to evaporate hexane. The residue was weighted, and the result was expressed as a percentage of powder (*n* = 3).

The encapsulation efficiency (EE) was calculated using Equation (1), where the total oil content refers to the amount of oil in the anhydrous fraction of prepared emulsion (30%), as calculated based on the emulsion formulation.

(1) $$\mathrm{EE}\;\left(\%\right)=\frac{\left(\mathrm{Total}\;\mathrm{oil}\;\mathrm{content}\;-\mathrm{Surface}\;\mathrm{oil}\;\mathrm{content}\right)}{\mathrm{Total}\;\mathrm{oil}\;\mathrm{content}}\;\cdot 100$$

#### 3.4.2. Particle Size and Particle Size Distribution

The particle size and particle size distribution of the powders were determined using laser diffraction with a Mastersizer 2000 (Malvern Instruments Ltd., Worcestershire, UK). An encapsulated powder was diluted 100-fold with isopropanol. The refractive index values of the dispersed phase and dispersant were 1.450 and 1.375, respectively. However, the absorption index had a value of 0.

The structure of the powders was characterized by the Sauter mean diameter D[3, 2], which is the surface-weighted mean diameter; the De Broucker mean diameter D[4, 3], which is the volume-weighted mean diameter; and the specific surface area (SSA). The mean sizes of the distribution, d(0.1), d(0.5), and d(0.9) (representing the diameters at which 10%, 50%, or 90% of the sample, respectively, has smaller particles than the measured size), were used to calculate the distribution width (Span) according to Equation (2) [73,74].

(2) $$span=\frac{d\left(0.9\right)-d(0.1)}{d(0.5)}$$

#### 3.4.3. Density and Flowing Properties

Bulk and tapped densities were determined according to GEA Niro Analytical Method A2a [75] using a jolting Stampf-volumeter (Engelsmann AG, Ludwigshafen am Rhein, Germany) with a 250 mL measuring glass cylinder. The powder sample in the cylinder was slightly tapped 10 times to remove the powder sticking to the walls (bulk density) or was tapped 100 times (tapped density) to measure the final volume. These density measurements were then used to calculate the Carr’s index [76] (as shown in Equation (3)).

(3) $$Carr’s\;index(\%)=\frac{Tapped\;density-Bulk\;density}{Tapped\;density}\cdot 100$$

#### 3.4.4. Water Activity

The water activity of the powder was determined by measuring the equilibrium relative humidity of the air above its surface in a closed chamber at a constant temperature (24 °C). The measurements were performed using a Rotronic (Warsaw, Poland) HC2A-AW-USB probe with a Rotronic AW-KHS locking handle and a Rotronic WP-40TH sample holder with a water jacket.

#### 3.4.5. Color and Morphology

The color of the samples was measured using digital image analysis (DIA) following the method described by Tańska et al. [77]. Results were expressed in the CIEL*a*b* color space model, where L* indicates lightness, a* represents the greenness/redness, and b* represents the blueness/yellowness.

The morphology of the powders was investigated using scanning electron microscopy (SEM) with a Quanta 200 microscope (FEI Company, Hillsboro, OR, USA), as described by Ogrodowska et al. [78]. Images were captured at a magnification of 400×.

#### 3.4.6. Oxidative Stability

The oxidative stability of the powders was determined using a Rancimat 743 apparatus (Metrohm, Herisau, Switzerland), based on the procedure described by Ogrodowska et al. [78]. Samples (2.5 g) were weighed into reaction vessels and heated at 110 °C. A continuous stream of air was passed through the samples at a flow rate of 20 L/h. The test measures the conductivity of the volatile compounds that are formed from the oxidation. The results were expressed as induction times in h.

### 3.5. Statistical Analysis

All analyses were performed at least in triplicate, and the results obtained were processed using Statistica 13.3 software (TIBCO, Palo Alto, CA, USA). One-way analysis of variance (ANOVA) was conducted, including Tukey’s test for homogenous groups. Additionally, two-factorial ANOVA was performed, and Pareto charts were generated using the same software.

## 4. Conclusions

The study showed that the emulsion type significantly influenced the functional properties of linseed oil powders. Nanoemulsification improved the encapsulation efficiency (by reducing surface oil content) and flow properties, as reflected by lower tapped density and Carr’s index, compared to microemulsification. Nanoemulsification also increased emulsion viscosity, particularly in formulations with nutriose. However, Rancimat test results revealed that powders derived from nanoemulsions exhibited lower oxidative stability, which was associated with their low surface oil content. This finding suggests that oxidative processes predominately occur in the non-encapsulated oil fraction. These results highlight the importance of optimizing encapsulation efficiency to mitigate the rate of oil oxidation. Additionally, the selection of wall material significantly impacted powder characteristics, with inulin producing the smallest and most uniform particles, regardless of the emulsion type. The study underscores the importance of adjusting both wall material composition and emulsion type to achieve desired powder properties. Further research into the mechanisms driving oxidative stability and their interaction with encapsulation parameters would provide deeper insights into industrial applications.

## Figures and Tables

**Table 1:** Encapsulation efficiency of linseed oil emulsions prepared with maltodextrin (1,2), nutriose (3,4), and inulin (5,6) using micro- (1,3,5) and nanoemulsification (2,4,6), and surface oil content of the obtained powders.

Sample Number	Surface Oil Content (%)	Encapsulation Efficiency (%)
1	3.13 ± 0.11 ^c^	89.57 ± 0.38 ^b^
2	0.36 ± 0.01 ^a^	98.82 ± 0.02 ^d^
3	4.62 ± 0.11 ^d^	84.62 ± 0.35 ^a^
4	0.29 ± 0.01 ^a^	99.03 ± 0.05 ^d^
5	2.08 ± 0.21 ^b^	93.08 ± 0.68 ^c^
6	0.44 ± 0.02 ^a^	98.55 ± 0.07 ^d^

^a–d^—mean values in columns marked with different superscript letters indicate significant differences (*p* < 0.05).

**Table 2:** Particle size, specific surface area (SSA), and Span of powders from linseed oil emulsions prepared with maltodextrin (1,2), nutriose (3,4), and inulin (5,6) using micro- (1,3,5) and nanoemulsification (2,4,6).

Sample Number	d(0.1) (μm)	d(0.5) (μm)	d(0.9) (μm)	D[3, 2] (μm)	D[4, 3] (μm)	SSA	Span
1	17.8 ± 0.1 ^b,c^	39.1 ± 0.8 ^c,d^	694.3 ± 171.0 ^b^	34.7 ± 0.9 ^c^	172.1 ± 25.5 ^c^	0.17 ± 0.01 ^b^	17.2 ± 4.0 ^b^
2	24.9 ± 0.1 ^e^	51.8 ± 0.8 ^e^	144.8 ± 22.1 ^a^	45.9 ± 0.8 ^d^	100.9 ± 17.4 ^b^	0.13 ± 0.00 ^a^	2.3 ± 0.4 ^a^
3	16.0 ± 0.8 ^a^	34.2 ± 0.8 ^a^	90.8 ± 3.7 ^a^	29.9 ± 0.9 ^a^	104.7 ± 8.5 ^b^	0.20 ± 0.01 ^c^	2.2 ± 0.2 ^a^
4	20.2 ± 0.2 ^d^	40.6 ± 0.8 ^d^	81.7 ± 1.4 ^a^	35.5 ± 0.2 ^c^	54.7 ± 7.8 ^a^	0.17 ± 0.00 ^b^	1.5 ± 0.0 ^a^
5	18.5 ± 0.5 ^c^	36.4 ± 1.2 ^a,b^	75.5 ± 4.1 ^a^	32.4 ± 1.1 ^b^	58.6 ± 8.5 ^a^	0.19 ± 0.01 ^c^	1.6 ± 0.0 ^a^
6	16.8 ± 0.0 ^a,b^	37.1 ± 0.2 ^b,c^	86.0 ± 2.2 ^a^	31.3 ± 0.2 ^a,b^	57.1 ± 4.7 ^a^	0.19 ± 0.00 ^c^	1.9 ± 0.0 ^a^

^a–e^—mean values in columns marked with different superscript letters indicate significant differences (*p* < 0.05).

**Table 3:** Water activity, density, and Carr’s index of powders from linseed oil emulsions prepared with maltodextrin (1,2), nutriose (3,4), and inulin (5,6) using micro- (1,3,5) and nanoemulsification (2,4,6).

Sample Number	Water Activity (%)	Bulk Density (g/cm^3^)	Tapped Density (g/cm^3^)	Carr’s Index (%)
1	0.27 ± 0.00 ^a^	0.256 ± 0.005 ^a,b^	0.506 ± 0.006 ^b,c^	49.50 ± 1.53 ^b,c^
2	0.31 ± 0.00 ^c^	0.264 ± 0.000 ^a,b^	0.488 ± 0.011 ^b^	45.76 ± 1.19 ^b,c^
3	0.26 ± 0.01 ^a^	0.257 ± 0.006 ^a,b^	0.486 ± 0.006 ^b^	47.05 ± 1.95 ^b,c^
4	0.26 ± 0.00 ^a^	0.248 ± 0.003 ^a^	0.417 ± 0.008 ^a^	40.46 ± 1.84 ^a^
5	0.30 ± 0.01 ^b^	0.267 ± 0.013 ^b^	0.533 ± 0.006 ^c^	49.96 ± 1.96 ^c^
6	0.31 ± 0.00 ^c^	0.289 ± 0.001 ^c^	0.524 ± 0.018 ^c^	44.82 ± 1.66 ^a,b^

^a–c^—mean values in columns marked with different superscript letters indicate significant differences (*p* < 0.05).

**Table 4:** Color of powders from linseed oil emulsions prepared with maltodextrin (1,2), nutriose (3,4), and inulin (5,6) using micro- (1,3,5) and nanoemulsification (2,4,6).

Sample Number	L* Parameter	a* Parameter	b* Parameter
1	98.31 ± 0.09 ^a^	−1.87 ± 0.03 ^b^	6.41 ± 0.11 ^b^
2	97.40 ± 1.62 ^a^	−1.87 ± 0.13 ^b^	5.89 ± 0.42 ^a,b^
3	98.60 ± 0.06 ^a^	−1.79 ± 0.02 ^b^	6.58 ± 0.01 ^b^
4	98.65 ± 0.15 ^a^	−1.83 ± 0.09 ^b^	5.86 ± 0.33 ^a,b^
5	96.66 ± 1.14 ^a^	−2.21 ± 0.17 ^a^	8.04 ± 0.60 ^c^
6	98.22 ± 0.19 ^a^	−1.61 ± 0.04 ^b^	5.07 ± 0.13 ^a^

^a–c^—mean values in columns marked with different superscript letters indicate significant differences (*p* < 0.05).

**Table 5:** Homogenization pressure and formulation of emulsions.

Sample	Homogenization Pressure (MPa)	Water (%)	Linseed Oil (%)	Maltodextrin (%)	Nutriose (%)	Inulin (%)	OSA starch (%)
1	24/4	70	10	10	-	-	10
2	150	70	10	10	-	-	10
3	24/4	70	10	-	10	-	10
4	150	70	10	-	10	-	10
5	24/4	70	10	-	-	10	10
6	150	70	10	-	-	10	10

## Data Availability

The raw data supporting the conclusions of this article will be made available by the authors on request.

## Supplementary Materials

The following supporting information can be downloaded at: https://www.mdpi.com/article/10.3390/molecules30010171/s1, Figure S1. Pareto charts generated by the variance analyses using ANOVA for analyzed variables.
