# Supplementary material for: Impact of Wall Material Composition (Maltodextrin vs. Inulin vs. Nutriose) and Emulsion Preparation System (Nano- vs. Microemulsion) on Properties of Spray-Dried Linseed Oil
Source: Molecules. 2025 Jan 4;30(1):171. doi: 10.3390/molecules30010171 (PMC11722179; doi:10.3390/molecules30010171)
Supplement: Supplementary file 1 [file molecules-30-00171-s001.zip › molecules-3363708-supplementary.pdf]

molecules

Supplementary material

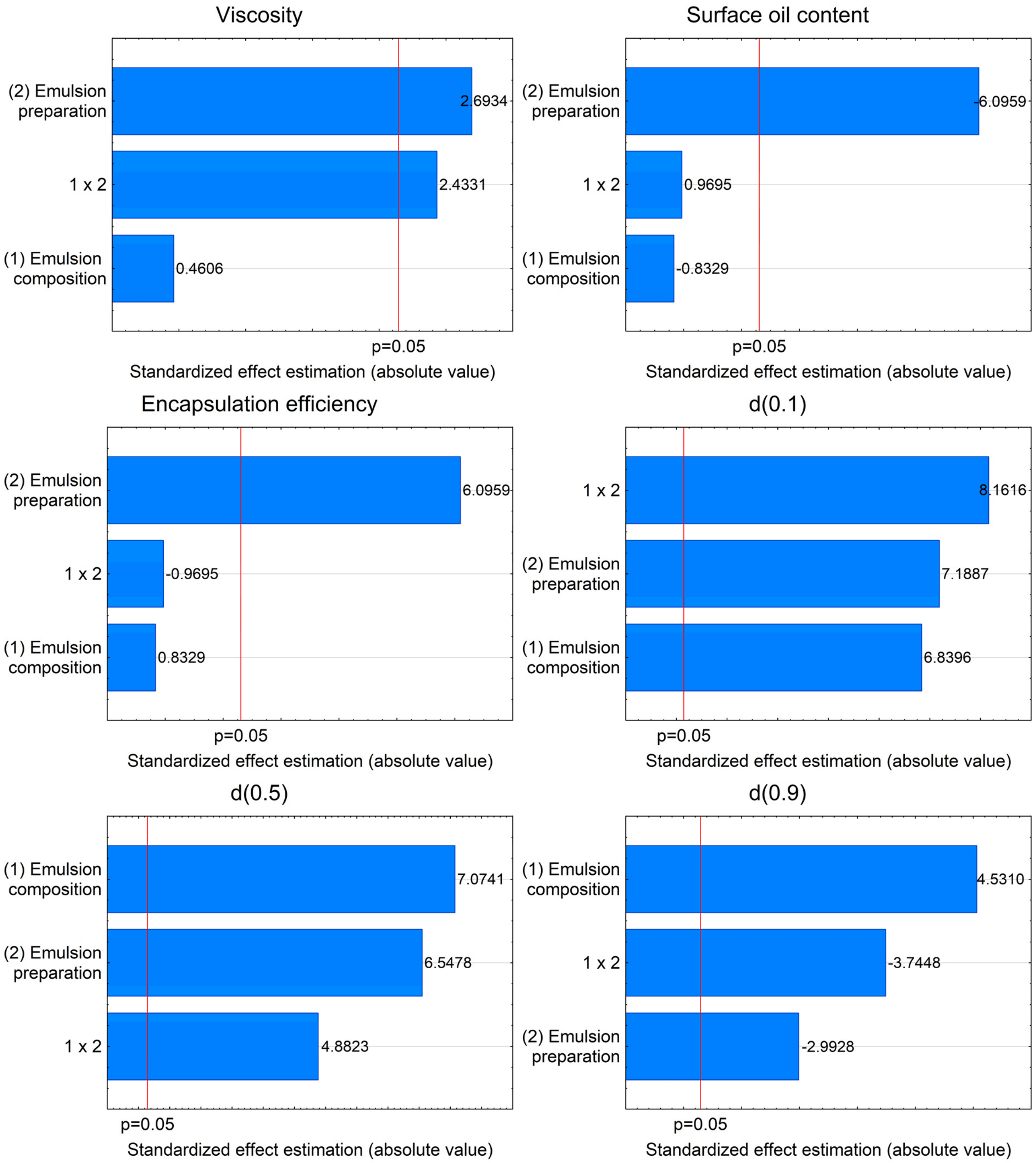

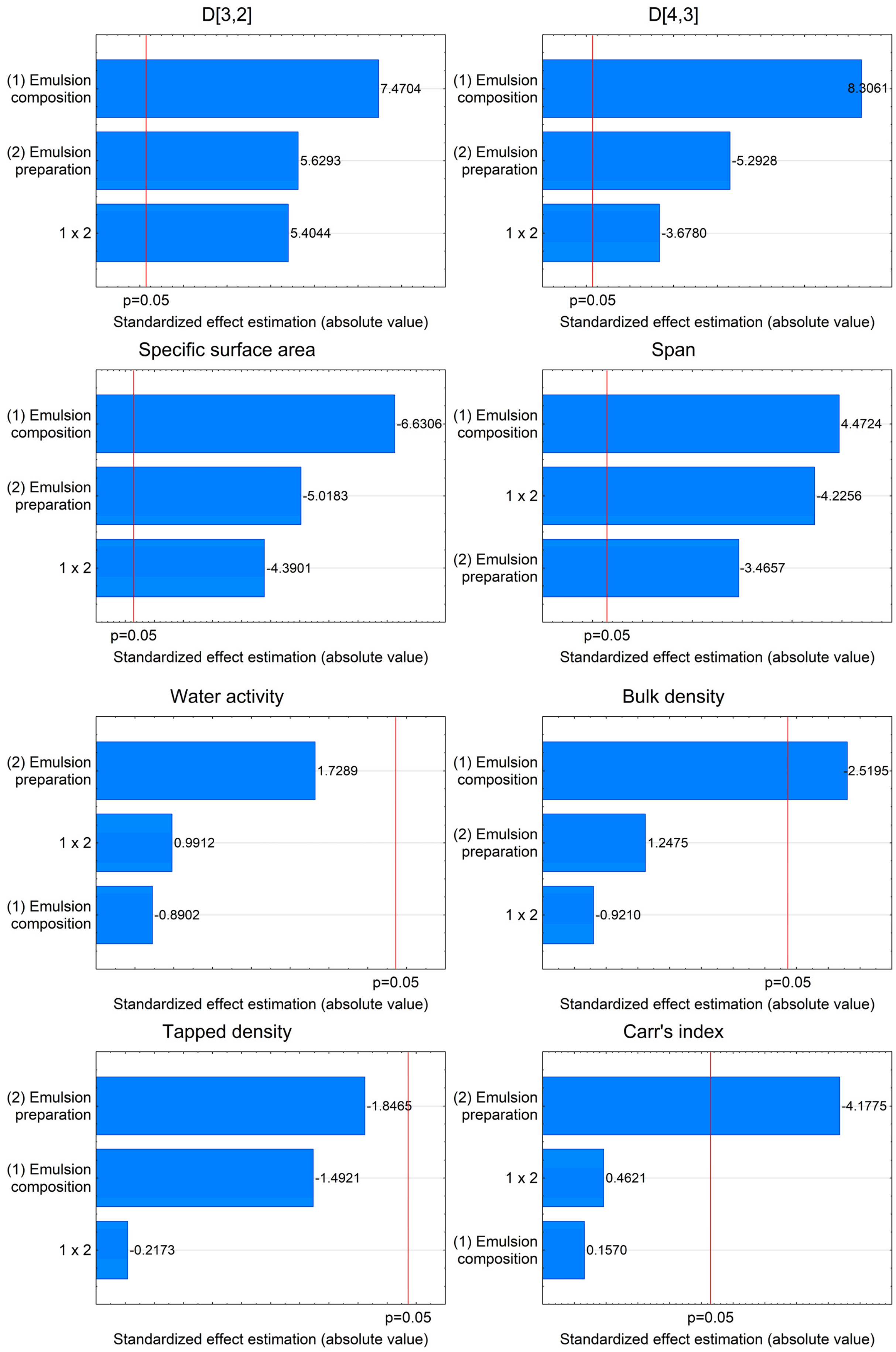
D[3,2]
(1) Emulsion composition
7.4704
(2) Emulsion preparation
5.6293
1 x 2
5.4044
p=0.05
Standardized effect estimation (absolute value)
D[4,3]
(1) Emulsion composition
8.3061
(2) Emulsion preparation
-5.2928
1 x 2
-3.6780
p=0.05
Standardized effect estimation (absolute value)
Specific surface area
(1) Emulsion composition
-6.6306
(2) Emulsion preparation
-5.0183
1 x 2
-4.3901
p=0.05
Standardized effect estimation (absolute value)
Span
(1) Emulsion composition
4.4724
1 x 2
-4.2256
(2) Emulsion preparation
-3.4657
p=0.05
Standardized effect estimation (absolute value)
Water activity
(2) Emulsion preparation
1.7289
1 x 2
0.9912
(1) Emulsion composition
-0.8902
p=0.05
Standardized effect estimation (absolute value)
Bulk density
(1) Emulsion composition
-2.5195
(2) Emulsion preparation
1.2475
1 x 2
-0.9210
p=0.05
Standardized effect estimation (absolute value)
Tapped density
(2) Emulsion preparation
-1.8465
(1) Emulsion composition
-1.4921
1 x 2
-0.2173
p=0.05
Standardized effect estimation (absolute value)
Carr's index
(2) Emulsion preparation
-4.1775
1 x 2
0.4621
(1) Emulsion composition
0.1570
p=0.05
Standardized effect estimation (absolute value)

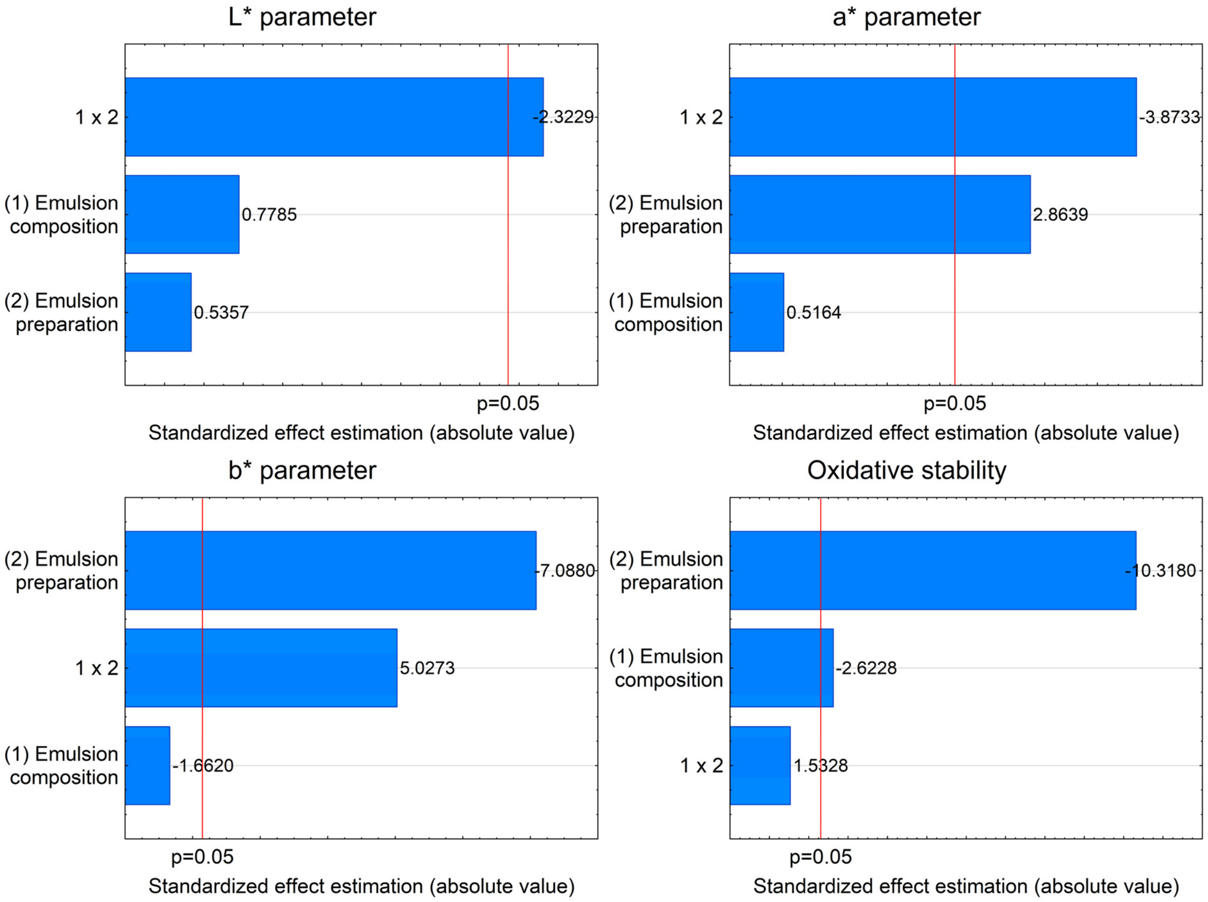

Figure S1. Pareto charts generated by the variance analyses using ANOVA for analyzed variables.
